# Identifying selection signatures for immune response and resilience to Aleutian disease in mink using genotype data

**DOI:** 10.3389/fgene.2024.1370891

**Published:** 2024-07-12

**Authors:** Guoyu Hu, Duy Ngoc Do, Ghader Manafiazar, Alyson A. Kelvin, Mehdi Sargolzaei, Graham Plastow, Zhiquan Wang, Pourya Davoudi, Younes Miar

**Affiliations:** ^1^ Department of Animal Science and Aquaculture, Dalhousie University, Truro, Canada; ^2^ Vaccine and Infectious Disease Organization (VIDO), University of Saskatchewan, Saskatoon, Canada; ^3^ Department of Pathobiology, University of Guelph, Guelph, Canada; ^4^ Select Sires Inc., Plain City, OH, United States; ^5^ Livestock Gentec, Department of Agricultural, Food and Nutritional Science, University of Alberta, Edmonton, Canada

**Keywords:** Aleutian disease resilience, American mink, selection signatures, genotypes, immune response

## Abstract

Aleutian disease (AD) brings tremendous financial losses to the mink industry. Selecting AD-resilient mink has been conducted to control AD. Such selections could have altered the patterns of genetic variation responding to selection pressures. This study aimed to identify selection signatures for immune response (IRE) and resilience to AD. A total of 1,411 mink from an AD-positive facility were used. For IRE, 264 animals were categorized according to the combined results of enzyme-linked immunosorbent assay (ELISA) and counterimmunoelectrophoresis (CIEP). For resilience, two grouping methods were used: 1) general resilience performance (GRP, n = 30) was evaluated based on the feed conversion ratio, Kleiber ratio, and pelt quality; and 2) female reproductive performance (FRP, n = 36) was measured based on the number of kits alive 24 h after birth. Detection methods were the pairwise fixation index, nucleotide diversity, and cross-population extended haplotype homozygosity. A total of 619, 569, and 526 SNPs were identified as candidates for IRE, GRP, and FRP, respectively. The annotated genes were involved in immune system process, growth, reproduction, and pigmentation. Two olfactory-related Gene Ontology (GO) terms were significant (q < 0.05) for all traits, suggesting the impact of AD on the sense of smell of infected mink. Differences in detected genes and GO terms among different color types for IRE indicated variations in immune response to AD among color types. The mitogen-activated protein kinase (MAPK) signaling pathway was significant (q < 0.05) for FRP, suggesting that AD may disrupt MAPK signaling and affect FRP. The findings of this research contribute to our knowledge of the genomic architecture and biological mechanisms underlying AD resilience in mink.

## 1 Introduction

Aleutian disease (AD) is one of the most severe diseases in mink farming, leading to significant financial losses to the mink industry due to its adverse influences on several economically important traits (Henson et al., 1962; Porter et al., 1982; Farid and Ferns, 2011; Reichert and Kostro, 2014). This disease is caused by the Aleutian mink disease virus (AMDV) and is defined as an immune-complex disease. Specific antibodies produced against AMDV fail to neutralize the virus and instead form complexes with the infectious virus, resulting in damage to the mink’s glomerular and arterial systems (Porter et al., 1969; Cho and Ingram, 1973; Porter et al., 1973; Stolze and Kaaden, 1987). Thus, the higher the levels of anti-AMDV antibodies produced, the more severe the infection caused by AMDV (Porter et al., 1972; Kanno et al., 1993; Bloom et al., 1994; Aasted et al., 1998; Bloom et al., 2001). Meanwhile, AMDV infection was also found to cause adverse influences on body weight growth (Porter et al., 1982), feed intake (Elzhov et al., 2016; Jensen et al., 2016), pelt quality (Farid and Ferns, 2011), and female reproductive performance (Henson et al., 1962; Reichert and Kostro, 2014). Thus, the anti-AMDV antibody level, growth, feed efficiency, and female reproductive performance were suggested as AD-resilience indicator traits in previous studies (Hu et al., 2021; Hu et al., 2022). Several methods, including vaccination, medicine, and culling strategy, have been attempted to control AD, but these methods have been largely ineffective. Consequently, mink farmers have resorted to selecting AD-resilient mink based on AD tests and/or AD-resilience indicator traits, such as body size, pelt quality, and reproductive performance, in AD-positive farms. Several mink farms in the Canadian province of Nova Scotia select AD-resilient mink using the iodine agglutination test and assessments of the productive performance (Farid and Ferns, 2017). Similarly, some findings indicated that some AD-positive mink farms in North America and Europe have applied enzyme-linked immunosorbent assay tests (ELISA) to select AD-resilient mink (Knuuttila et al., 2009; Farid and Rupasinghe, 2016; Farid et al., 2018).

Selection could cause changes in the patterns of genetic variation among selected loci and linked neutral loci (Kreitman, 2000; Qanbari and Simianer, 2014; Ma et al., 2015). These patterns are termed selection signatures, and they can be used to identify loci subject to the selection (Kreitman, 2000; Qanbari and Simianer, 2014; Ma et al., 2015). Therefore, identifying the signatures would be helpful in detecting genes and biological processes related to AD resilience. Characterizing the genomic regions associated with mink response to AD could aid in the development of breeding programs focusing on improving AD-resilience in mink farms. Several statistical methods have been proposed for detecting selection signatures in livestock. The pairwise fixation index (Fst) (Weir and Cockerham, 1984), nucleotide diversity (θπ) (Nei and Li, 1979), and cross-population extended haplotype homozygosity (XP-EHH) (Sabeti et al., 2007) are the three methods commonly used to detect selection signatures, where Fst and θπ are based on the genetic diversity and genetic differentiation, and XP-EHH is based on the frequency of extended haplotypes between two subpopulations.

Advancements in next-generation sequencing (NGS) technologies, high-density single-nucleotide polymorphism (SNP) arrays, and bioinformatics tools have now significantly improved the detection of selection signatures in livestock species (Bertolini et al., 2018). For example, studies using selection signatures have identified several genes associated with disease resistance/susceptibility in cattle (Li et al., 2020; Saravanan et al., 2021). Xu et al. (2020) conducted a signature of selection study and detected several genes related to the susceptibility of swine to respiratory disease. For AD in American mink, Karimi et al. (2021) detected 99 genomic regions associated with the response to AMDV infection using genotyping by sequencing (GBS) data and five phenotypes (the anti-AMDV antibody titer, mortality, AD symptoms in the kidneys, and virus clearance at two different times) from 225 experimental black American mink that were inoculated with AMDV. These regions encompassed 63 genes involved in immune response, liver development, and reproduction (Karimi et al., 2021).

The ineffectiveness of vaccination, medication, and culling strategies in controlling AD has compelled farmers to select AD-resilient mink (Knuuttila et al., 2009; Farid and Rupasinghe, 2016; Farid and Ferns, 2017; Farid et al., 2018). However, the absence of a comprehensive understanding of the genetic/genomic architecture of AD resilience hinders breeders from incorporating this innovative trait into their breeding programs. In previous studies (Hu et al., 2021; Hu et al., 2022), the genetic and phenotypic parameters for various AD tests and other AD-resilient or economically important traits in mink, including reproductive performance, growth, feed intake, and pelt quality, were elucidated. The findings delineated the heritabilities of AD tests and other AD-resilient traits and genetic relationships among them. The outcomes also emphasized the antigen-based enzyme-linked immunosorbent assay test (ELISA-G) as the most reliable and practical indicator trait for selecting AD-resilient mink among all AD tests (Hu et al., 2021; Hu et al., 2022). The population genomic study conducted by Hu et al. (2023) investigated the genetic structures of farmed mink in Canada with diverse color types using the genotypes. The findings updated the linkage disequilibrium patterns and the effective population size of studied populations and revealed genetic distance, genetic diversity, and the admixture pattern of studied populations. The population genomic information from the study provided the essential information to implement the SNP panel in genomic studies of American mink. However, the signature of selection study focusing on the response of mink to AD had not been conducted using genotype data in conjunction with AD-resilience indicator traits (e.g., growth, feed efficiency, pelt quality, and reproduction) for mink reared in AD-positive commercial farms. It has been reported that mink with darker color types seem to be more resilient to AMDV than lighter color types (Ellis, 1996). A signature of the selection study is a potential approach to study the performances of different color types of mink against AMDV infection. Therefore, the objectives of this study were to use genotype data and different color types of American mink to 1) detect the selection signatures associated with immune response, general resilience, and female reproductive performance resilience to AD; 2) identify the genes related to immune response, general resilience, and female reproductive performance resilience to AD; and 3) investigate whether mink of different color types exhibit distinct respond to AD and explore potential differences in AD resilience mechanisms among color variations.

## 2 Materials and methods

This study was approved by the Dalhousie University Animal Care and Use Committee (certification#: 2018-009 and 2019-012). All the mink were farmed following the Code of Practice for the Care and Handling of Farmed Mink guidelines from the Canada Mink Breeders Association (Turner P et al., 2013). The study was carried out in compliance with the ARRIVE 2.0 guidelines (Percie du Sert et al., 2020).

### 2.1 Animals and sampling

All the individuals (n = 1,411) studied in this research were from the Canadian Center for Fur Animal Research (CCFAR) at Dalhousie University, Faculty of Agriculture (Truro, Nova Scotia, Canada), from 2013 to 2021. In 2013, an outbreak of AD occurred at CCFAR, resulting in most of the mink being dead or culled in the barn. The exact source of the outbreak was not determined definitively, but it was suspected that AMDV-contaminated feed and contact with wild animals carrying AMDV were the most likely causes. Thus, within 3 years of the disease outbreak, approximately 150 mink (120 dam and 30 sires) from six AD-positive farms in Nova Scotia (Canada), which were believed to be resilient to AD and have been phenotypically selected for AD-resilient mink for many years, were introduced and used as breeders at CCFAR. AD was first identified in the province of Nova Scotia in Canada in 1941 (Agriculture_and_Marketing_of_Nova_Scotia_Government, 1998), and many mink farms in the province started selecting AD-resilient mink based on specific AD-resilient traits (Farid and Ferns, 2017). The studied mink included five different color types: black (n = 177), demi (n = 542), mahogany (n = 527), pastel (n = 152), and stardust (n = 13). The color type was identified by experienced technicians at CCFAR at weaning.

### 2.2 Aleutian disease test

Counterimmunoelectrophoresis (CIEP) and antigen-based enzyme-linked immunosorbent assay tests (ELISA-G) were used to measure the immune response of the studied mink to AMDV exposure. The tests were conducted using established protocols described by Hu et al. (2021). In brief, blood samples of the studied mink were collected in mid-November before selecting breeders and in mid-February before mating from 2013 to 2021. The blood samples were sent to the Animal Health Laboratory at the University of Guelph (Ontario, Canada) and Middleton Veterinary Services (Nova Scotia, Canada) for CIEP and ELISA-G tests, respectively. The CIEP tests were used to detect the existence of anti-AMDV antibodies, and the results were recorded as 0 (negative: none or extremely low antibody level detected) or 1 (positive: detectable antibody level). The ELISA-G tests were applied to measure amounts of antibodies against AMDV using optical density, and the test results included eight categories with 1-point increments from 0 (none or an extremely low level of antibodies) to 7 (extremely high antibody level).

### 2.3 Growth and measurement

The Kleiber ratio (KR) and feed conversion ratio (FCR) were used to measure the growth and feed efficiency of the studied mink, respectively. The body weights of the studied individuals were collected using the established protocols described by Do et al. (2021). In brief, the body weight (BW) of the mink was measured at both birth and weaning (around 6 weeks after birth) and every 3 weeks from 13 to 28 weeks after birth. The average daily gain (ADG) and mid-test metabolic BW (BW^0.75^) were calculated by the following equations, respectively:

$$ADG=\frac{Final\;BW-Initial\;BW}{\text{Number}\;\text{of}\;\text{days}\;\text{on}\;\text{the}\;\text{test}},$$

$${{BW}^{0.75}=\left(\frac{Initial\;BW+Final\;BW}{2}\right)}^{0.75},$$

where final BW was the BW on the last day of the feeding trial and the initial BW was the BW at 13 weeks of age. The Kleiber ratio (KR) was calculated using the following equation:

$$KR=\frac{ADG}{{BW}^{0.75}}.$$

The feed intake data on the studied mink were collected using the established protocols described by Davoudi et al. (2022). In brief, mink were raised individually in separate cages, and feed was distributed daily to cages. The daily feed intake (DFI) of each mink was measured by calculating the difference between the amount of feed left over and the feed provided. The individual DFI records obtained during the experiment were averaged to obtain the individual average daily feed intake (ADFI). The FCR was calculated using the following equation:

$$FCR=\frac{ADFI}{ADG}\;.$$

### 2.4 Pelt quality evaluation

The pelt quality tests were conducted using the same method described by Hu et al. (2021). The live grading of overall pelt quality (QUA) was performed to measure the qualities of the mink pelt when they were alive. The grading was conducted based on the North American Fur Auctions live animal grading procedure by a skilled technician. The grading focused on checking the color consistency, fur roughness, and overall gloss. The QUA was scored into three categories from 1 (poor) to 3 (best).

### 2.5 Female reproductive performance measurement

The female reproductive performance was measured using the same approach described by Hu et al. (2021). Female reproductive performance was measured and recorded by the technicians in CCFAR during each annual reproduction cycle from 2006 to 2021. In this study, the number of newborn kits that survived 24 h after birth was used to quantify the reproduction performance of dams under AMDV exposure.

### 2.6 Animal grouping

Studied individuals were grouped into pairwise subgroups based on their immune response, general resilience, and female reproductive performance. Studied individuals with both CIEP and ELISA-G test results were grouped into low- or high-immune response subgroups based on their CIEP and ELISA-G test results. Individuals with zero ELISA-G scores and negative CIEP results were grouped into low-immune response subgroups, and individuals with 5–7 ELISA-G scores and positive CIEP results were grouped into the high-immune response subgroup (Table 1). In this study, we not only grouped the entire populations of individuals into low- or high-immune response subgroups but also the individuals within the same color type, which included black, demi, mahogany, and pastel color types (Table 1). For resilience indicator traits, two methods were used to group CIEP-positive individuals into pairwise groups (Table 2). Studied individuals with positive CIEP results that had BW, feed intake, and pelt quality records were grouped into resilient or susceptible subgroups. The CIEP-positive individuals, which had bottom 20% FCR (14.38–22.49), top 20% KR (7.14–9.17), and score 3 (high pelt quality) for QUA were grouped into the resilient subgroup, and the CIEP-positive individuals, which had top 20% FCR (41.46–72.28), bottom 20% KR (2.19–4.41), and score 1 (low pelt quality) for QUA, were grouped into the susceptible subgroup (Table 2). Meanwhile, studied CIEP-positive dams, which had records for the number of newborn kits that survived 24 h after birth, were grouped into low- or high-female reproductive performance subgroups. The CIEP-positive dams with less than four newborn kits that survived 24 h after birth were grouped into the low-reproductive performance subgroup, and the CIEP-positive dams that had more than nine newborn kits that survived 24 h after birth were grouped into the high-reproductive performance subgroup (Table 2).

**TABLE 1:** Number of individuals from different color types in each subgroup in different Aleutian disease tests and the final dataset for detecting selection signatures for immune response to Aleutian mink disease virus infection in American mink.

	ELISA-G^a^ (0-7)		CIEP^b^		Immune response^c^	
Color type	Negative (0)	Positive (5-7)	Negative	Positive	Low	High
Black	67	23	12	78	10	19
Demi	264	57	87	329	70	51
Mahogany	244	40	50	258	39	37
Pastel	42	31	14	70	11	25
Stardust	5	3	1	8	1^d^	1^d^
All	622	154	164	743	131	133

^a^ ELISA-G = AMDV-G-based enzyme-linked immunosorbent assay test.

^b^ CIEP = counterimmunoelectrophoresis test.

^c^ The individuals were used in the final dataset for detecting selection signatures for immune response to Aleutian mink disease virus infection.

^d^ No independent analysis was conducted for stardust color-type individuals due to the small sample size.

**TABLE 2:** Number of individuals with positive counterimmunoelectrophoresis test results in each subgroup of the feed conversion ratio, Kleiber ratio, live pelt quality, general resilience performance, and female reproductive performance traits.

Feed conversion ratio		Kleiber ratio		Pelt quality		General resilience performance^a^		Female reproductive performance^b^	
Bottom 20% (14.38–22.49)	Top 20% (41.46–72.28)	Bottom 20% (2.19–4.41)	Top 20% (7.14–9.17)	Score 1	Score 3	Resilient	Susceptible	Low litter size (1–4)	High litter size (9–11)
78	78	78	78	83	120	19	11	20	16

^a^ The evaluation of individual general resilience performance based on the feed conversion ratio, Kleiber ratio, and pelt quality.

^b^ The measurement of female reproductive performance resilience (dams with a positive counterimmunoelectrophoresis test only) based on the number of kits alive 24 h after birth.

### 2.7 Sample collection and genotype detection

Tongue tissues from studied individuals were collected before pelting. The DNeasy Blood and Tissue Kit (QIAGEN, Hilden, Germany) was used to extract the DNA from the tongue tissue based on the manufacturer’s instructions. The NanoDrop ND-1000 spectrophotometer (NanoDrop Technologies Inc., Wilmington, DE) was applied to measure the quantity and quality of the extracted DNA samples. The 260/280-nm readings for all samples ranged from 1.7 to 2.0. All samples had a final concentration of 20 ng and were finally genotyped by an Axiom Affymetrix Mink 70K SNP panel (Neogen, Lincoln, Nebraska, USA) (Do et al., 2024).

### 2.8 Animals and SNP quality control

PLINK (Purcell et al., 2007) was used to conduct animal and SNP data quality control. SNPs which had a minor allele frequency lower than 1%, call rate lower than 90%, excess of heterozygosity higher than 15%, Mendelian error frequency larger than 5%, and were out of Hardy–Weinberg equilibrium with a very low probability (1 × 10^−5^) were excluded from the analyses. Meanwhile, mink, which had a call rate lower than 90%, were also removed from the dataset. After quality control, 26,406 SNPs and 1,411 animals remained for further analyses.

### 2.9 Methods for the detection of selection signatures

Three methods, including the pairwise fixation index (Fst) (Weir and Cockerham, 1984), nucleotide diversity (θπ) (Nei and Li, 1979), and cross-population extended haplotype homozygosity (XP-EHH) (Sabeti et al., 2007), were performed to detect the selection signatures. The Fst and θπ methods directly utilize the SNP genotype, while the XP-EHH method uses phased data. The Fst analysis was conducted for each SNP based on the method proposed by Weir and Cockerham (1984) using VCFtools software (Danecek et al., 2011). The Z-transformation was performed using the *scale* function in the R program (Team, 2022) to normalize the Fst values. All negative Fst values were set to zero. The Fst values of all SNPs were ranked, and the SNPs with the top 5% Fst values were considered candidate selection signatures. The θπ analysis was conducted for each SNP based on the method proposed by Nei and Li (1979) using VCFtools software (Danecek et al., 2011). The θπ ratios were computed as θπ (subgroup1)/θπ (subgroup2) for all pairs of groups and were then log2-transformed (log2 (θπ ratios)). The SNPs with the top 5% θπ ratio values were considered candidate selection signatures. The XP-EHH approach was calculated for each SNP using selscan software (Torres et al., 2018). The missing genotypes were removed using VCFtools software (Danecek et al., 2011), and the genotypes were phased using Beagle software (Browning et al., 2018) because selscan software can only handle the phased genotypes without missing genotypes. The original obtained XP-EHH values were normalized using the norm function within the selscan software program. Then, the *pnorm* function in the R program was applied to calculate the *p*-values of the normalized XP-EHH values. The *p*-values of the normalized XP-EHH values were log-transformed, and the SNPs with -log (*p*-value) more than two were considered candidate selection signatures. Only the SNPs detected as candidate selection signatures by at least two methods were used for genome annotation, Gene Ontology, and functional analysis.

### 2.10 Genome annotation, Gene Ontology, and functional analysis

The potential selection regions were defined by extending 350 kb both upstream and downstream of the candidate selection signatures. The regions were defined based on the previous study that suggested that linkage disequilibrium (*r* ^2^ < 0.2) in the current studied American mink population did not exceed 350 kb (Hu et al., 2023). Genome annotation was conducted using bedtools software (Quinlan, 2014) referring to the genome assembly of *Neogale vison* (Karimi et al., 2022). The Gene Ontology (GO) terms, including biological process (GO:BP), cellular component (GO:CC), and molecular function (GO:MF), were assigned to annotated genes using PANTHER 14.1 (Thomas et al., 2003). The overrepresentation tests of annotated genes were conducted using Fisher’s exact test and adjusted by the false discovery rate (FDR) correction, and the terms with the FDR-adjusted *p*-value (q-value) <0.05 were considered the overrepresented terms. Meanwhile, the Kyoto Encyclopedia of Genes and Genomes (KEGG) pathway analyses were conducted using the *clusterProfiler* package (Yu et al., 2012) in the R program with FDR control.

## 3 Results

### 3.1 Selection signatures for the immune response trait

**FIGURE 1:**
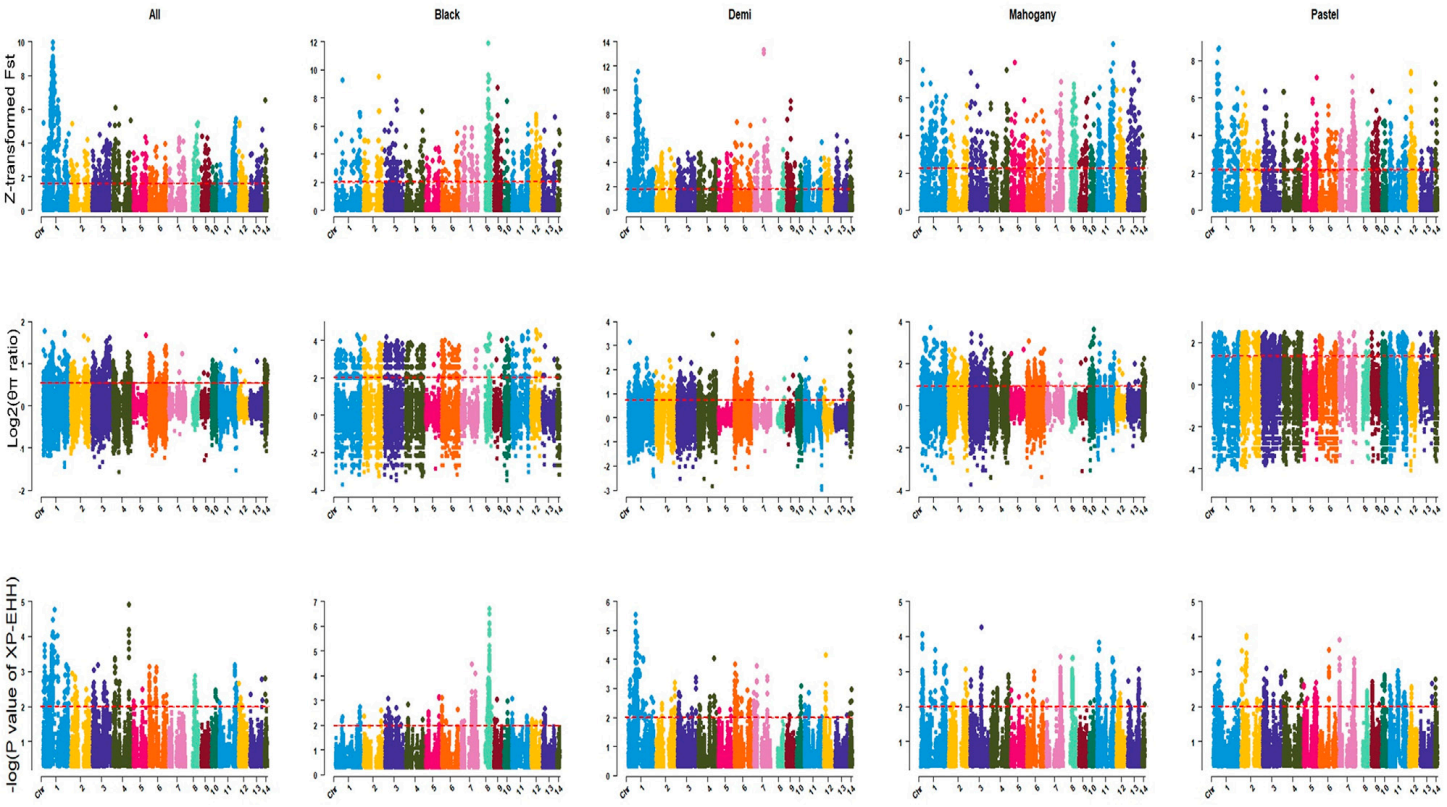
Genome-wide distribution of detected selection signatures for immune response trait across all autosomes in the whole population and different color types of individuals. The red lines of Z-transformed Fst and log2 (θπ ratio) plots display the threshold levels of 5%. The red lines of XP-EHH plots display the threshold levels of -log (*p*-value)>2.

**FIGURE 2:**
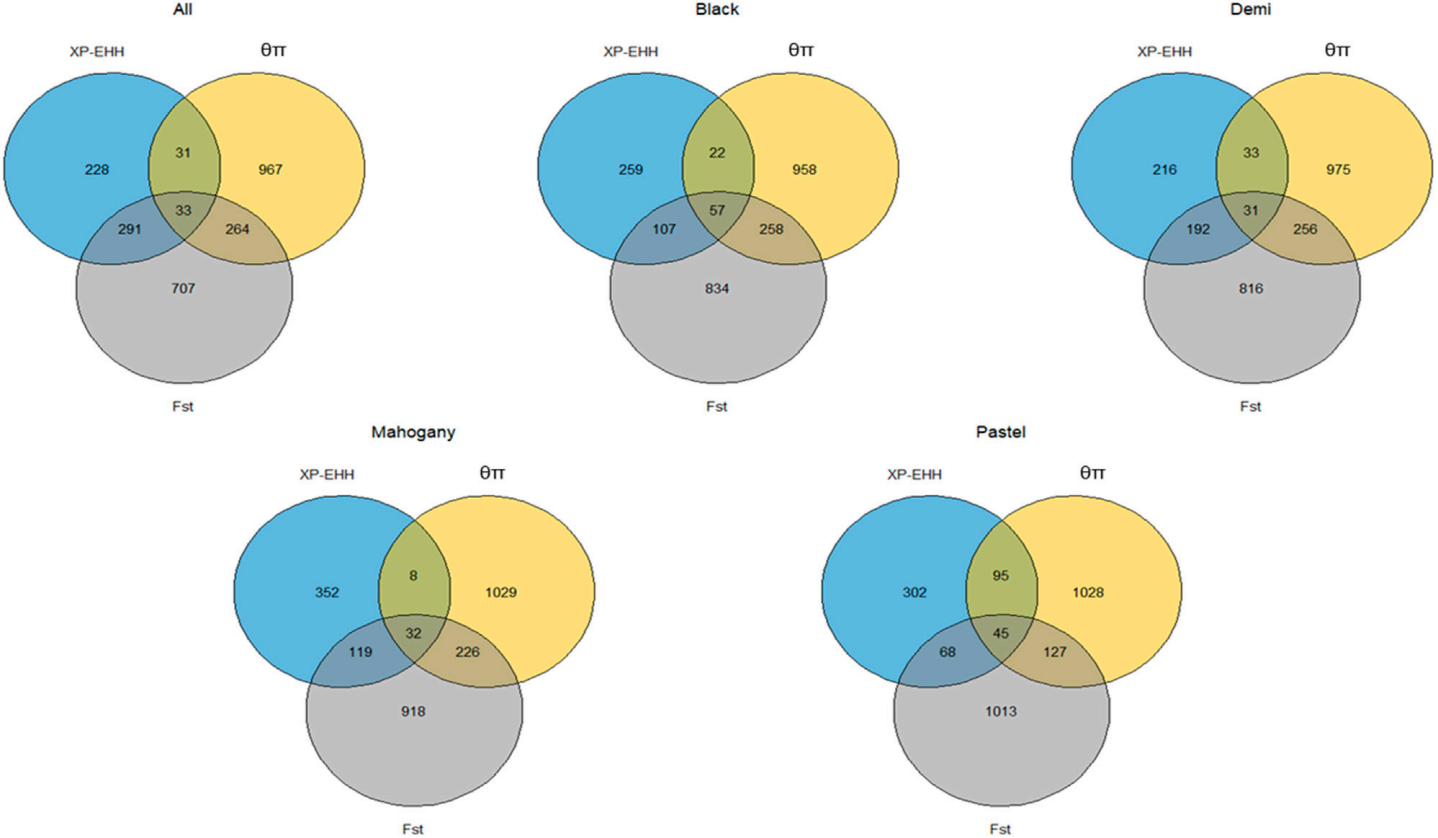
Overlapped selection signatures detected in the whole population and different color types of individuals among three detection methods for the immune response trait.

The genome-wide distribution of selection signatures associated with the immune response trait is presented in Figure 1. Additionally, Figure 2 illustrates the selection signatures that overlap across the three methods. Supplementary Material S1 presents the candidate selection signatures (chromosome number and location on the chromosome) detected by each method. When considering the entire population of individuals, a total of 619 SNPs were detected as candidate selection signatures by at least two methods. Notably, 33 SNPs were detected by all three methods and were considered strongly selected candidates (Figure 2). Furthermore, when analyzing individuals within specific color types, 444, 512, 385, and 335 were detected as candidate selection signatures for black, demi, mahogany, and pastel color-type mink, respectively. In addition, 57, 31, 32, and 45 SNPs were detected by all three methods for black, demi, mahogany, and pastel color-type mink, respectively, highlighting strong selection signature candidates specific to the immune response trait within each color type (Figure 2).

**FIGURE 3:**
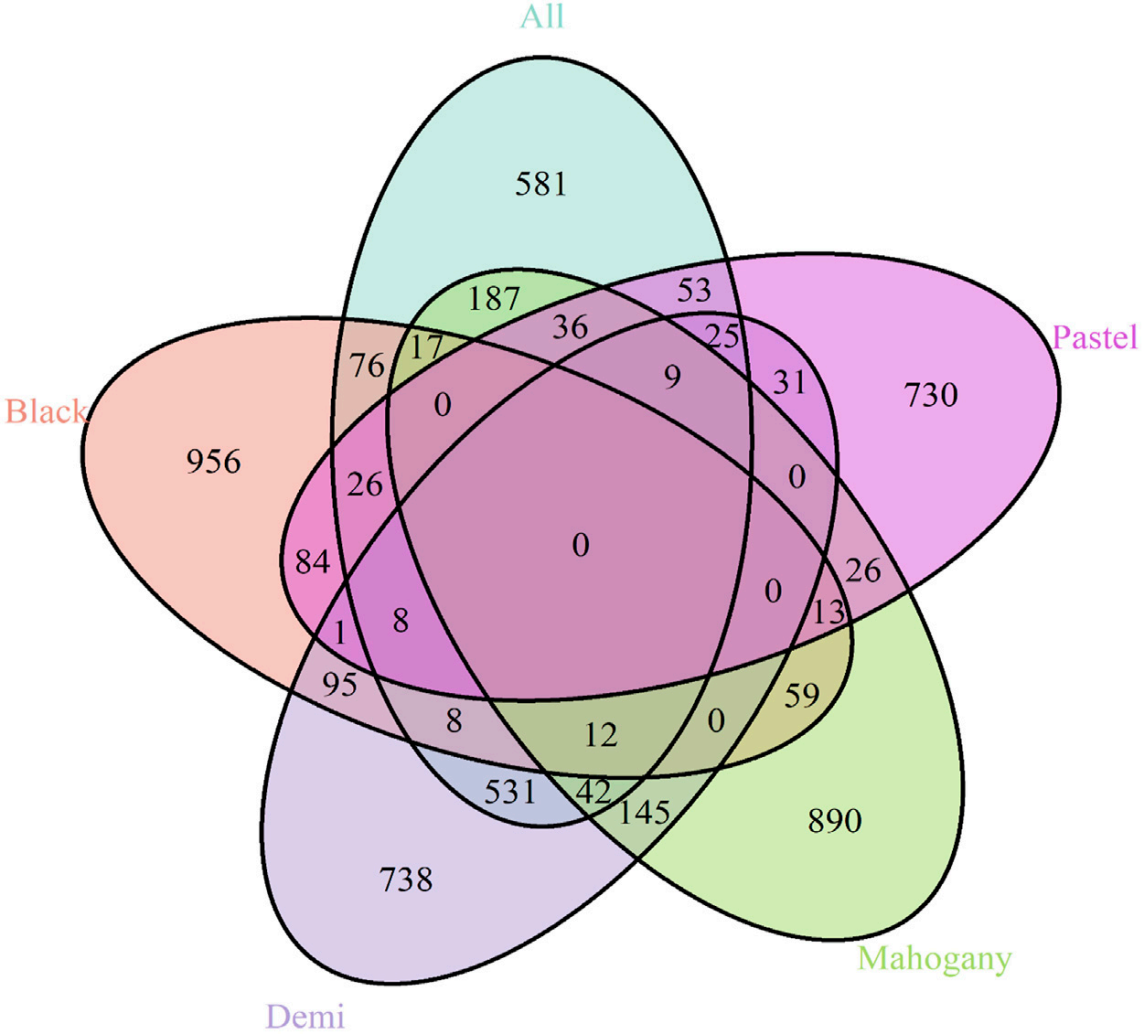
Venn diagram shows the genes overlapping among the whole population and different color types for the immune response trait.

**FIGURE 4:**
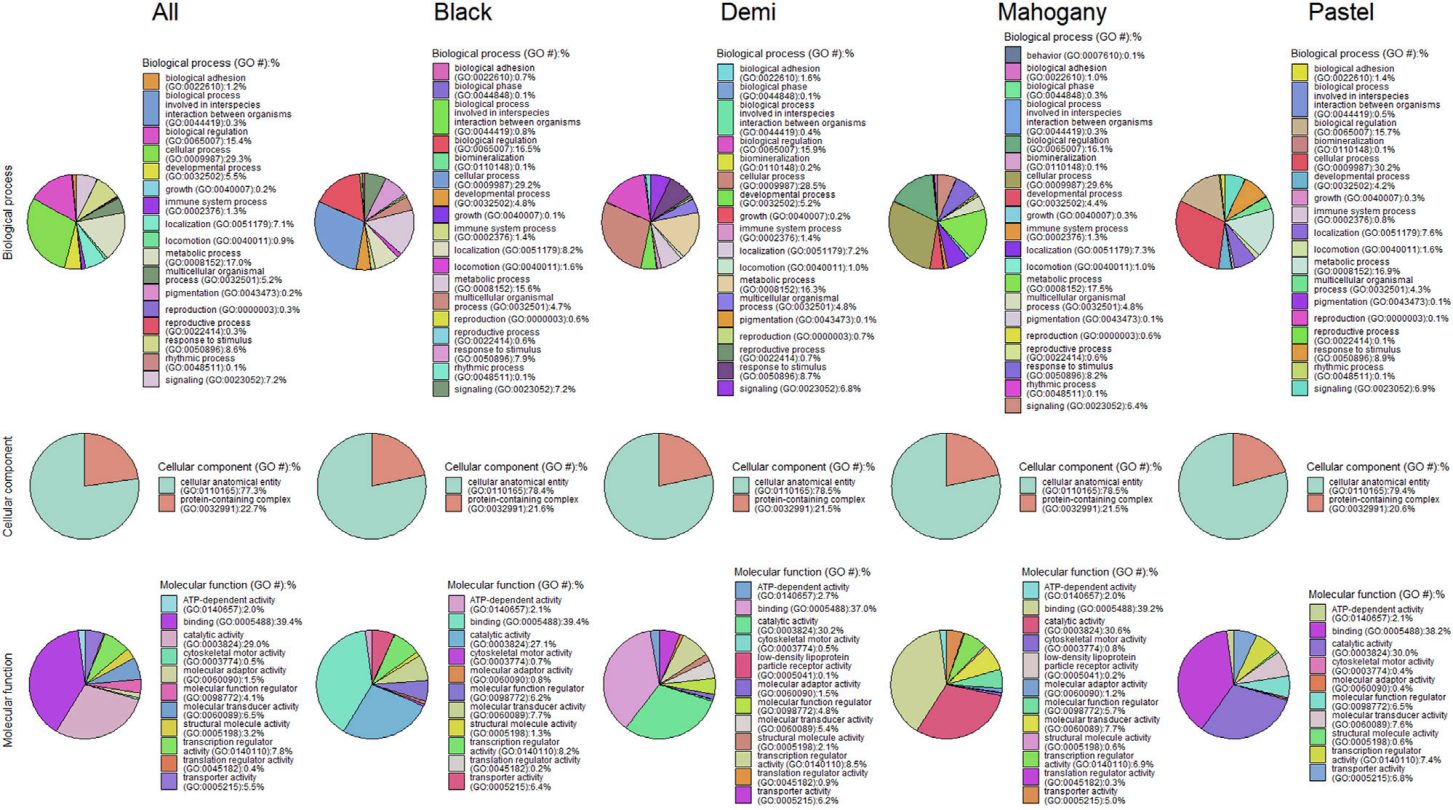
Pie charts of functional classifications of candidate genes under selection pressure in the whole population and different color types of individuals for immune response traits.

The candidate genes annotated from the selection signatures in the whole population and different color types are listed in Supplementary Material S1. Figure 3 shows the overlapped annotated candidate genes among the whole population and different color types. A total of 1,611 candidate genes were annotated from the selection signatures detected from the whole population (Figure 3 and Supplementary Material S2A). For black, demi, mahogany, and pastel color-type individuals, 1,355, 1,645, 1,436, and 1,042 candidate genes were annotated, respectively (Figure 3). Among the candidate genes annotated from the whole population and different color types, many genes were found to be associated with the AD-characterized phenotypes, including the immune system process, growth, pigmentation (except for the black color-type), reproduction, and response to stimulus (Supplementary Material S2, Figures 3, 4). Table 3 provides a list of genes for all color types. However, no common gene was detected among all color types for immune response traits (Figure 3).

**TABLE 3:** Immune response-related genes annotated from selection signatures detected from different color types.

Color type	Genes
Black	*ANKRD17*, *CEBPA*, *CTSH*, *CXCL6*, *CXCL8*, *FYB1*, *GAL*, *HAVCR1*, *IL1A*, *IL1F10*, *IL1RN*, *IL36RN*, *ITK*, *MARCHF1*, *MTURN*, *PATZ1*, *SIGLEC15*, *TMEM178A*, *TNFRSF1B*, and *UBASH3A*
Demi	*C4A*, *CACTIN*, *CCL26*, *CCR9*, *CEBPA*, *CGAS*, *CTLA4*, *CXCR6*, *DEF6*, *FYN*, *HIC2*, *HSPD1*, *MPIG6B*, *NFAM1*, *RUNX1*, *RUNX2*, *SH2B2*, *SHFL*, *TNFRSF21*, *TRAF3IP2*, *TYK2*, *VEGFA*, *XCR1*, *YES1*, *ZBTB12*, and *ZBTB37*
Mahogany	*ANKRD17*, *C4A*, *CASP3*, *CXCL6*, *CXCL8*, *EPOR*, *FYB2*, *HSPD1*, *MEIS1*, *MPIG6B*, *NFAM1*, *PLA2G2D*, *PLA2G2F*, *PLA2G5*, *RAG2*, *REL*, *TNFRSF11A*, *TNFRSF13C*, and *ZBTB12*
Pastel	*AKIRIN1*, *BANK1*, *BCL10*, *UBASH3A*, *FGR*, *LPXN*, *SEC14L1*, *THEMIS2*, and *TNFRSF1B*

Figure 4 presents the functional classifications of candidate genes related to the immune response trait. The genes annotated from the whole population, black, demi, mahogany, and pastel color-type individuals were classified into 17, 18, 17, 20, and 18 GO:BP categories, respectively, where the top four biological processes were the cellular process, metabolic process, biological regulation, and response to stimulus in all cases (Figure 4). The cellular anatomical entity and protein-containing complex were the two cellular components detected from the whole population and all color types. Regarding the GO:MF classifications, both analyses for whole population individuals and black color-type individuals detected 11 GO:MF, while analyses for demi, mahogany, and pastel color-type individuals detected 12 GO:MF. The top four GO:MF categories for the whole population and all color types were binding activity, catalytic activity, transcription regulator activity, and molecular transducer activity (Figure 4).

Table 4 (GO:BP) and Table 5 (GO:CC and GO:MF) present the overrepresentations of candidate genes. A total of 27, 18, 50, 26, and 18 significant (q-value<0.05) overrepresented GO enrichment terms were detected from the whole population, black, demi, mahogany, and pastel color-type individuals, respectively (Tables 4 and 5). Among all detected significant (q-value<0.05) overrepresented GO enrichment terms, some of them were commonly detected in the whole population and all color types (Tables 4 and 5), including two GO:BP (detection of chemical stimulus involved in sensory perception of smell (GO:0050911) and sensory perception of smell (GO:0007608)), three GO:CC (cytoplasm (GO:0005737), intracellular anatomical structure (GO:0005622), and organelle (GO:0043226)), and one molecular function (olfactory receptor activity (GO:0004984)). However, some were only detected in a certain color type of mink or the entire population. For example, the biological processes of adaptive immune response (GO:0002250) and system development (GO:0048731) were only detected for the whole population. Several metabolism-related GO:BP (e.g., heterocycle metabolic process (GO:0046483), macromolecule metabolic process (GO:0043170), and nitrogen compound metabolic process (GO:0006807)) and several GO:CC (e.g., envelope (GO:0031975), protein-containing complex (GO:0032991), and synapse (GO:0045202)) were only detected in demi color-type mink. Two unique GO:MF, namely, catalytic activity (GO:0003824) and hydrolase activity (GO:0016787), were only detected in mahogany color-type mink.

**TABLE 4:** Significant (q-value<0.05) biological processes detected from overrepresentation tests of candidate genes from the whole studied population and different color types of mink for immune response traits.

Biological process (GO ID)	All	Black	Demi	Mahogany	Pastel
Adaptive immune response (GO:0002250)	a	b	b	b	b
Anatomical structure development (GO:0048856)	a	b	a	a	a
Anatomical structure morphogenesis (GO:0009653)	a	b	b	a	b
Bicellular tight junction assembly (GO:0070830)	b	b	a	b	b
Cell development (GO:0048468)	a	b	a	a	b
Cell differentiation (GO:0030154)	a	b	a	a	b
Cellular aromatic compound metabolic process (GO:0006725)	b	b	a	b	b
Cellular component morphogenesis (GO:0032989)	b	b	b	a	b
Cellular component organization (GO:0016043)	b	b	a	a	b
Cellular component organization or biogenesis (GO:0071840)	b	b	a	a	b
Cellular developmental process (GO:0048869)	a	b	a	a	b
Cellular metabolic process (GO:0044237)	b	b	a	b	b
Cellular nitrogen compound metabolic process (GO:0034641)	b	b	a	b	b
Detection of chemical stimulus (GO:0009593)	a	a	a	b	a
Detection of chemical stimulus involved in sensory perception (GO:0050907)	a	a	a	b	a
Detection of chemical stimulus involved in sensory perception of smell (GO:0050911)	a	a	a	a	a
Detection of stimulus (GO:0051606)	a	b	b	b	b
Detection of stimulus involved in sensory perception (GO:0050906)	a	a	b	b	b
Developmental process (GO:0032502)	a	b	a	a	b
Heterocycle metabolic process (GO:0046483)	b	b	a	b	b
Macromolecule metabolic process (GO:0043170)	b	b	a	a	b
Metabolic process (GO:0008152)	b	b	a	b	b
Mitochondrial gene expression (GO:0140053)	b	b	a	b	a
Multicellular organism development (GO:0007275)	a	b	b	a	a
Multicellular organismal process (GO:0032501)	a	b	b	b	b
Nitrogen compound metabolic process (GO:0006807)	b	b	a	b	b
Nucleobase-containing compound metabolic process (GO:0006139)	b	b	a	b	b
Organelle organization (GO:0006996)	b	b	b	a	b
Organic substance metabolic process (GO:0071704)	b	a	a	a	b
Positive regulation of the multicellular organismal process (GO:0051240)	b	b	b	b	a
Primary metabolic process (GO:0044238)	b	b	a	a	b
Regulation of the multicellular organismal process (GO:0051239)	b	b	b	b	a
Sensory perception of chemical stimulus (GO:0007606)	a	a	a	b	a
Sensory perception of smell (GO:0007608)	a	a	a	a	a
Small-molecule catabolic process (GO:0044282)	b	a	b	b	b
System development (GO:0048731)	a	b	b	b	b
Tight junction organization (GO:0120193)	b	b	a	b	b

a The biological process was detected in this color type/population.

b The biological process was not detected in this color type/population.

**TABLE 5:** Significant (q-value<0.05) cellular components and molecular functions detected from overrepresentation tests of candidate genes from the whole studied population and different color types of mink for immune response traits.

Functional enrichment item	All	Black	Demi	Mahogany	Pastel
Cellular component (GO ID)					
Bicellular tight junction (GO:0005923)	b	b	a	b	b
Cell junction (GO:0030054)	a	b	a	b	b
Cellular anatomical entity (GO:0110165)	b	a	a	b	a
Collagen type IV trimer (GO:0005587)	b	b	b	b	a
Cytoplasm (GO:0005737)	a	a	a	a	a
Cytosol (GO:0005829)	b	a	a	b	b
Endomembrane system (GO:0012505)	b	b	b	b	a
Envelope (GO:0031975)	b	b	a	b	b
Intracellular anatomical structure (GO:0005622)	a	a	a	a	a
Intracellular membrane-bounded organelle (GO:0043231)	b	a	a	a	b
Intracellular organelle (GO:0043229)	b	a	a	a	a
Intracellular organelle lumen (GO:0070013)	b	b	a	b	b
Membrane-bounded organelle (GO:0043227)	b	a	a	a	a
Membrane-enclosed lumen (GO:0031974)	b	b	a	b	b
Mitochondrial matrix (GO:0005759)	b	b	a	b	b
Mitochondrion (GO:0005739)	b	b	a	b	b
Organelle (GO:0043226)	a	a	a	a	a
Organelle envelope (GO:0031967)	b	b	a	b	b
Organelle lumen (GO:0043233)	b	b	a	b	b
Protein-containing complex (GO:0032991)	b	b	a	b	b
Synapse (GO:0045202)	b	b	a	b	b
Tight junction (GO:0070160)	b	b	a	b	b
Molecular function (GO ID)					
Actin binding (GO:0003779)	a	b	b	b	b
Binding (GO:0005488)	a	a	a	a	b
Catalytic activity (GO:0003824)	b	b	b	a	b
Hydrolase activity (GO:0016787)	b	b	b	a	b
Identical protein binding (GO:0042802)	b	b	a	b	b
Olfactory receptor activity (GO:0004984)	a	a	a	a	a
Protein binding (GO:0005515)	a	a	a	a	b
Protein-containing complex binding (GO:0044877)	a	b	b	b	b
RNA binding (GO:0003723)	b	b	a	b	b
RNA polymerase II-specific DNA-binding transcription factor binding (GO:0061629)	a	b	b	b	b

^a^ The cellular component or molecular function was detected in this color type/population.

^b^ The cellular component or molecular function was not detected in this color type/population.

### 3.2 Selection signatures for general resilience and female reproductive performance traits

**FIGURE 5:**
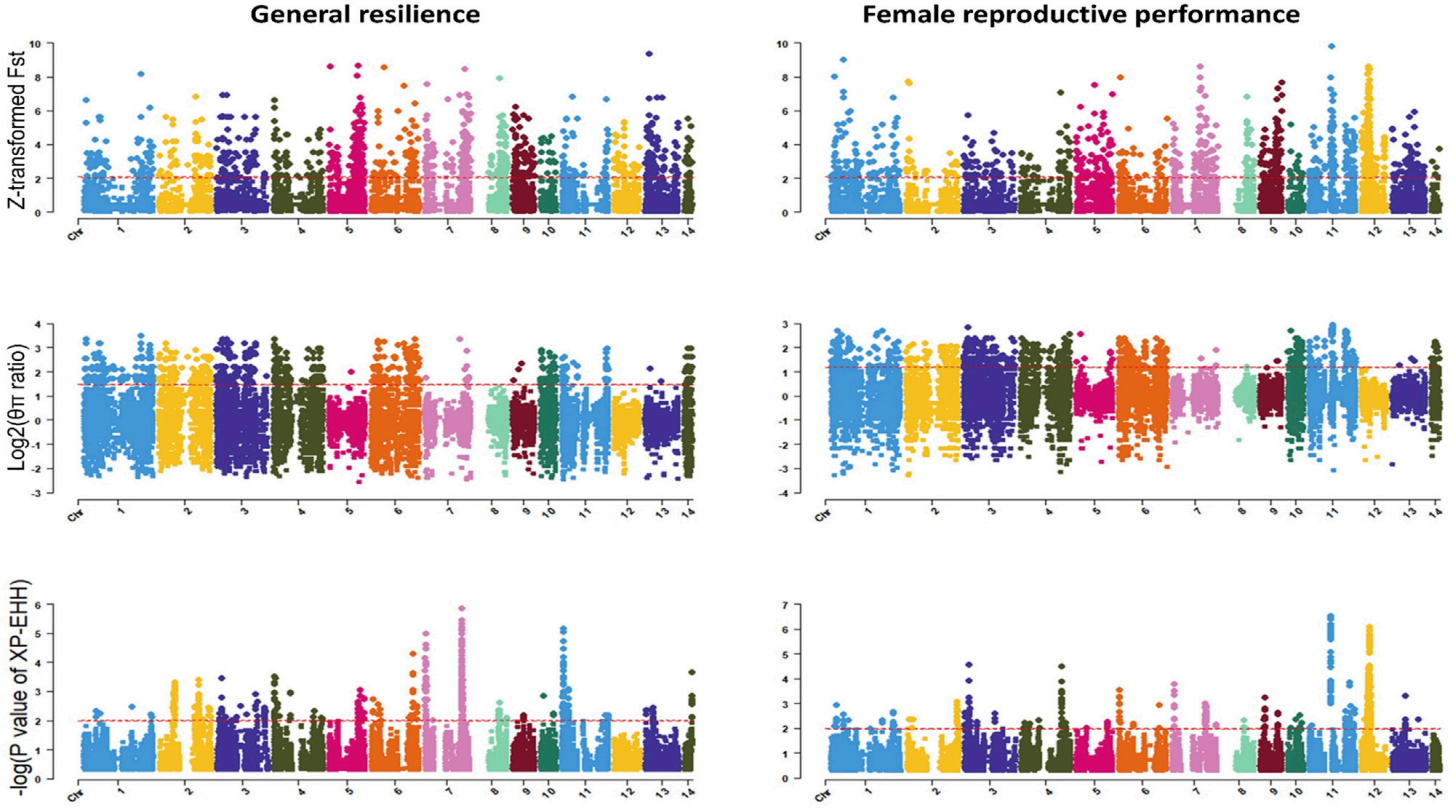
Genome-wide distribution of detected selection signatures for general resilience and female reproductive performance traits across all autosomes from the whole population. The red lines of Z-transformed Fst and log2 (θπ ratio) plots display the threshold levels of 5%. The red lines of XP-EHH plots display the threshold levels of -log (*p*-value)>2.

**FIGURE 6:**
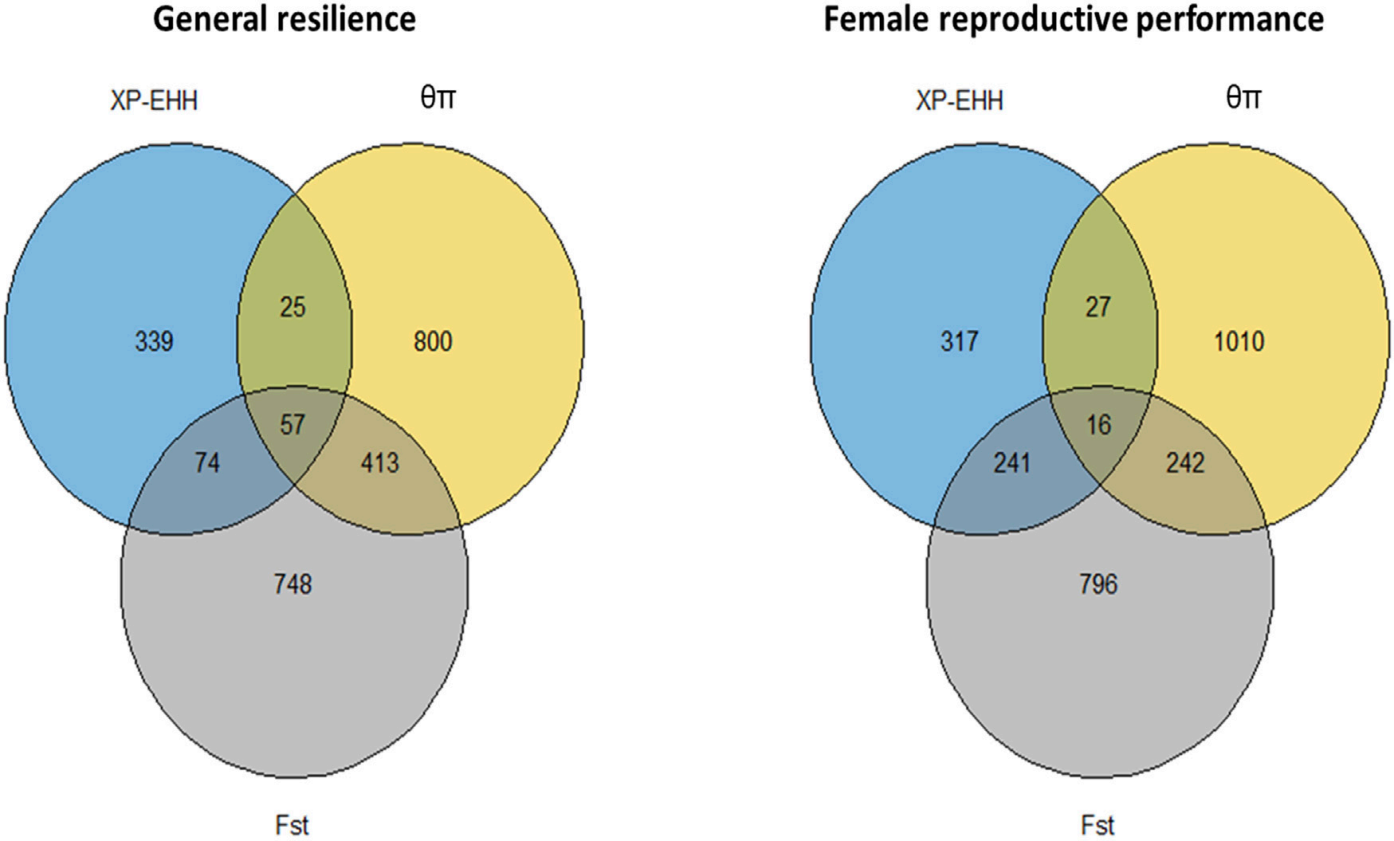
Overlapped selection signatures detected from the whole population individuals for general resilience and female reproductive performance traits.

The genome-wide distribution of selection signatures for general resilience and female reproductive performance traits are presented in Figure 5, and the overlapped selection signatures are presented in Figure 6. Supplementary Material S3 presents the SNPs detected as candidate selection signatures by each method and the SNPs detected as candidate selection signatures by at least two methods. For general resilience traits, 569 SNPs were detected as candidate selection signatures by at least two methods, and 57 SNPs were detected as candidate selection signatures by all three methods (Figure 6). For female reproductive performance traits, 526 SNPs were detected as candidate selection signatures by at least two methods, and 16 SNPs were detected as candidate selection signatures by all three methods (Figure 6).

**FIGURE 7:**
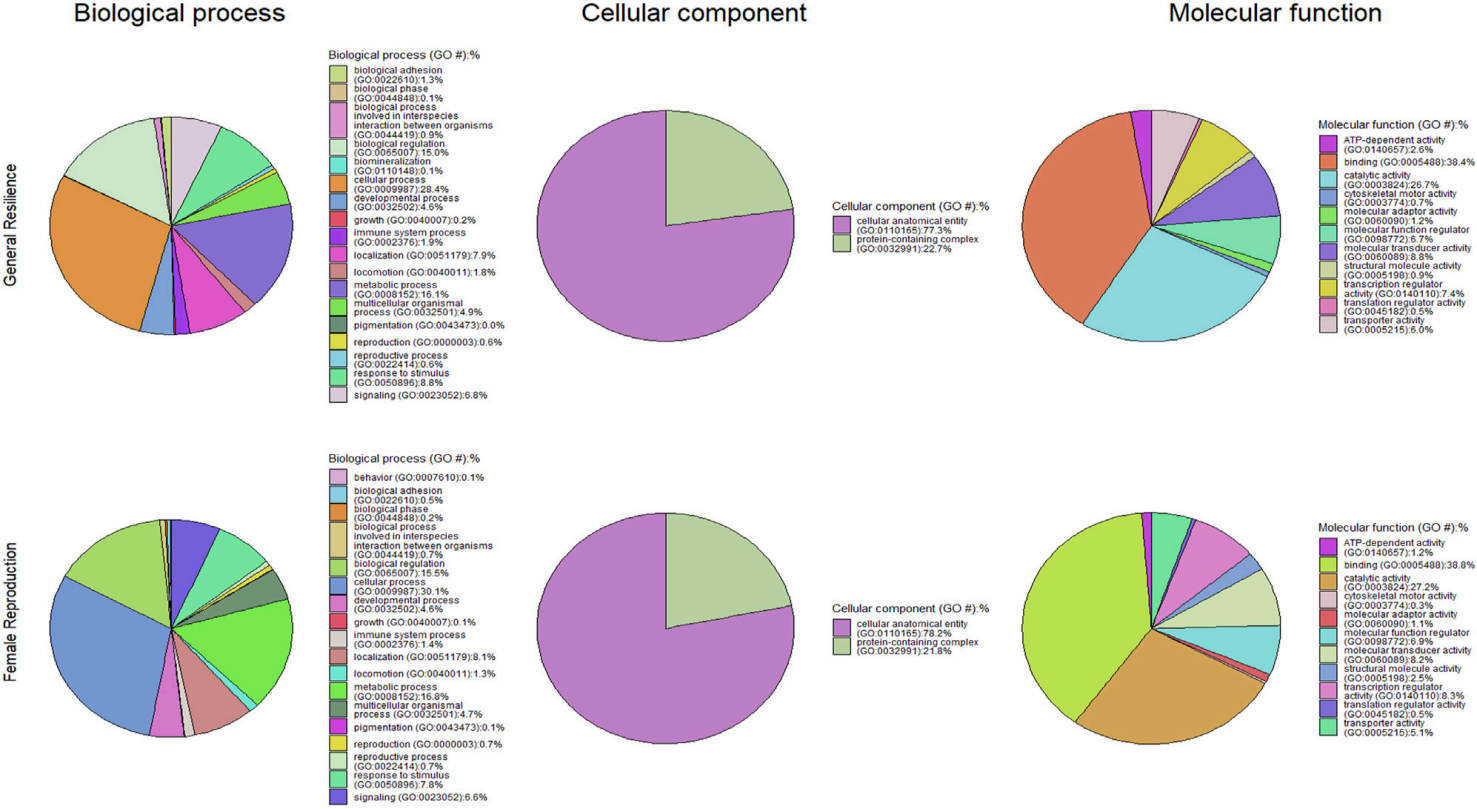
Pie charts of functional classifications of candidate genes under selection pressure in the whole population individuals for general resilience and female reproductive performance traits.

The candidate genes annotated from the candidate selection are listed in Supplementary Material S3, and the functional classifications of candidate genes are shown in Figure 7. A total of 1,933 genes were annotated from the selection signatures for the general resilience trait (Supplementary Material S2B). Several annotated genes were related to AD resilient traits, including growth, immune system process, pigmentation, and reproduction. The functional classifications of the annotated genes resulted in 18 GO:BP, 2 GO:CC, and 11 GO:MF. The annotation of selection signatures related to the female reproductive performance trait resulted in a total of 1,538 genes (Supplementary Material S2B). Except for 10 genes related to reproduction, several other genes were related to some AD-resilience indicator traits, including growth, immune system process, and pigmentation. The annotated genes were classified into 18 GO:BP, 2 GO:CC, and 11 GO:MF.

The overrepresentations of candidate genes are presented in Table 6. For general resilience traits, nine significant (q-value<0.05) overrepresented GO:BP were detected, and they were involved primarily in development, cellular process, and sensory perception of smell. Meanwhile, nine GO:CC (mostly related to organelle) and three GO:MF (related to olfactory receptor activity or binding) were significant (q-value<0.05) in the overrepresentation tests of candidate genes related to the general resilience. The overrepresentation tests of candidate genes from female reproductive performance traits resulted in 10 significant (q-value<0.05) overrepresented GO:BP (mostly related to development and detection of stimulus), 6 significant overrepresented GO:CC (mostly related organelle), and 4 significant overrepresented GO:MF (related to olfactory receptor activity or binding).

**TABLE 6:** Significant (q-value<0.05) biological processes, cellular components, and molecular functions detected from overrepresentation tests of candidate genes for general resilience and female reproductive performance traits.

Trait	Term (GO ID)	Annotation set
General resilience	Anatomical structure morphogenesis (GO:0009653)	Biological process
	Cell migration (GO:0016477)	Biological process
	Cell motility (GO:0048870)	Biological process
	Cellular process (GO:0009987)	Biological process
	Chemotaxis (GO:0006935)	Biological process
	Detection of chemical stimulus involved in sensory perception of smell (GO:0050911)	Biological process
	Locomotion (GO:0040011)	Biological process
	Sensory perception of smell (GO:0007608)	Biological process
	Taxis (GO:0042330)	Biological process
	Cell projection (GO:0042995)	Cellular component
	Cellular anatomical entity (GO:0110165)	Cellular component
	Cytoplasm (GO:0005737)	Cellular component
	Intracellular anatomical structure (GO:0005622)	Cellular component
	Intracellular membrane-bounded organelle (GO:0043231)	Cellular component
	Intracellular organelle (GO:0043229)	Cellular component
	Membrane-bounded organelle (GO:0043227)	Cellular component
	Organelle (GO:0043226)	Cellular component
	Plasma membrane-bounded cell projection (GO:0120025)	Cellular component
	Binding (GO:0005488)	Molecular function
	Olfactory receptor activity (GO:0004984)	Molecular function
	Protein binding (GO:0005515)	Molecular function
Female reproductive performance	Adaptive immune response (GO:0002250)	Biological process
	Anatomical structure development (GO:0048856)	Biological process
	Cellular process (GO:0009987)	Biological process
	Detection of chemical stimulus (GO:0009593)	Biological process
	Detection of chemical stimulus involved in sensory perception (GO:0050907)	Biological process
	Detection of chemical stimulus involved in sensory perception of smell (GO:0050911)	Biological process
	Developmental process (GO:0032502)	Biological process
	Protein metabolic process (GO:0019538)	Biological process
	Sensory perception of chemical stimulus (GO:0007606)	Biological process
	Sensory perception of smell (GO:0007608)	Biological process
	Cellular anatomical entity (GO:0110165)	Cellular component
	Cytoplasm (GO:0005737)	Cellular component
	Intracellular anatomical structure (GO:0005622)	Cellular component
	Intracellular membrane-bounded organelle (GO:0043231)	Cellular component
	Membrane-bounded organelle (GO:0043227)	Cellular component
	Organelle (GO:0043226)	Cellular component
	Binding (GO:0005488)	Molecular function
	Olfactory receptor activity (GO:0004984)	Molecular function
	Protein binding (GO:0005515)	Molecular function
	Protein–arginine deiminase activity (GO:0004668)	Molecular function

### 3.3 Common genes among studied traits and KEGG pathways

**FIGURE 8:**
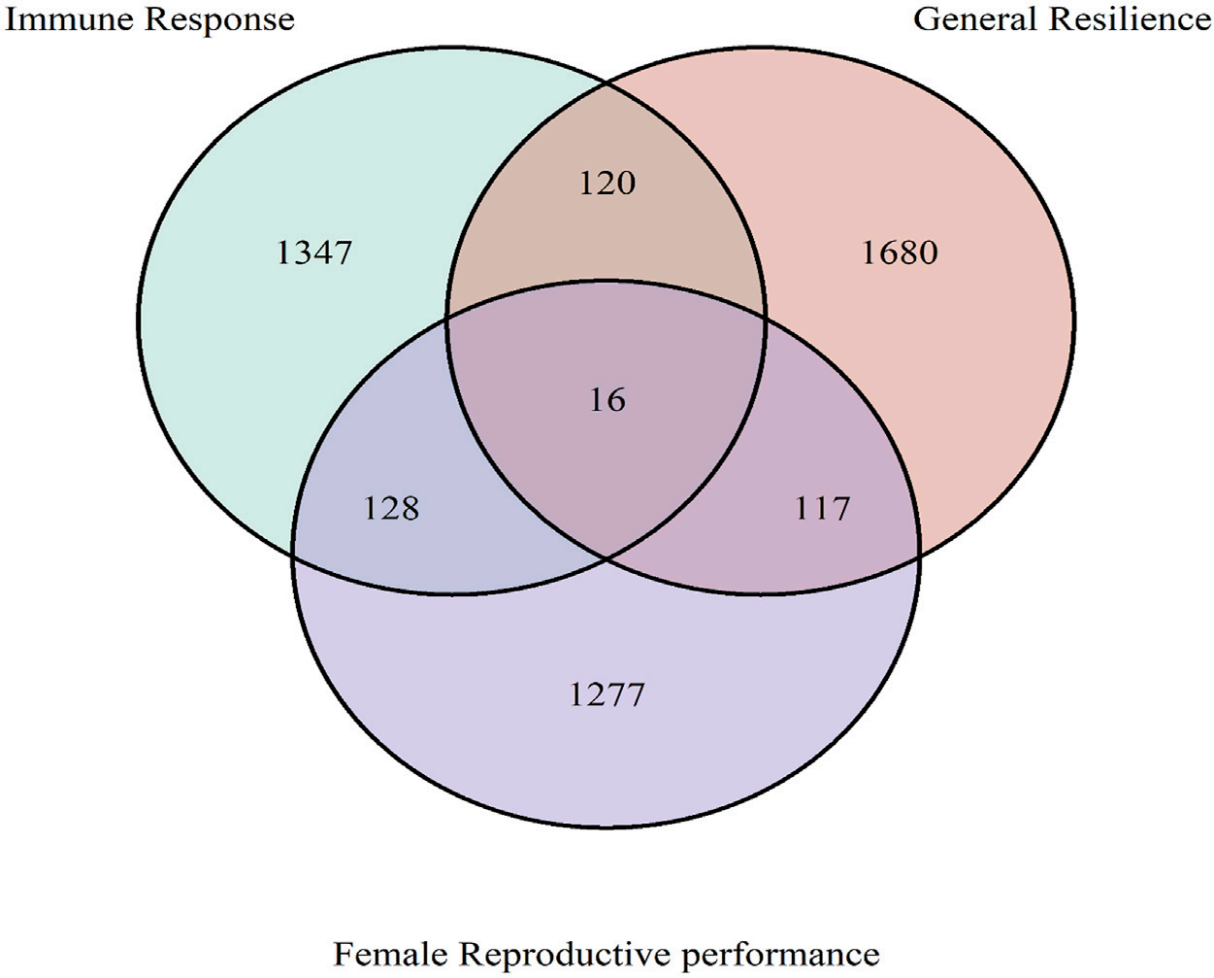
Overlapped genes among immune response, general resilience, and female reproductive performance traits. The common 16 genes among all traits were *ARHGAP19*, *COL14A1*, *DEPTOR, EXOSC1*, *FAM135B*, *FRAT1*, *FRAT2*, *LOC122905718*, *MMS19*, *MRPL13*, *PGAM1*, *PTCHD4*, *RRP12*, *TBX18*, *UBTD1*, and *ZDHHC16.*

The overlapped genes among immune response, general resilience, and female reproductive performance traits are presented in Figure 8. In brief, 1,347, 1,680, and 1,277 unique genes were annotated from the selection signatures related to immune response, general resilience, and female reproductive performance traits, respectively. Sixteen genes, namely, *ARHGAP19* (chr2: 209,800,177–209,812,754 bp), *COL14A1* (chr4: 19,536,084–19,755,397 bp), *DEPTOR* (chr4: 19,803,040–19,933,557 bp), *EXOSC1* (chr2: 209,917,603–209,928,479 bp), *FAM135B* (chr4: 4,922,384–4,961,329 bp), *FRAT1* (chr2: 209,829,018–209,831,565 bp), *FRAT2* (chr2: 209,839,939–209,842,216 bp), *LOC122905718* (chr4: 5,215,903–5,216,010 bp), *MMS19* (chr2: 209,938,058-209,971,536 bp), *MRPL13* (chr4: 19,501,767–19,518,965 bp), *PGAM1* (chr2: 209,910,223–209,917,518 bp), *PTCHD4* (chr1: 94,047,590–94,230,349 bp), *RRP12* (chr2: 209,866,295–209,895,940 bp), *TBX18* (chr1: 44,912,005–44,922,875 bp), *UBTD1* (chr2: 209,972,082–210,024,677 bp), and *ZDHHC16* (chr2: 209,928,631–209,937,825 bp), were detected from all three studied traits.

The significant (q < 0.05) KEGG pathways of candidate genes from immune response, general resilience, and female reproductive performance traits are listed in Table 7. For immune response traits, only one significant (q < 0.05) pathway, the longevity regulating pathway, was detected. For female reproductive performance traits, one significant (q < 0.05) pathway, the mitogen-activated protein kinase (MAPK) signaling pathway, was detected. No significant (q < 0.05) KEGG pathway was detected for the general resilience trait.

**TABLE 7:** Significantly (q-value<0.05) presented Kyoto Encyclopedia of Genes and Genomes (KEGG) pathways of genes detected from signature selection analyses of the immune response, general resilience, and female reproductive performance traits.

Trait	Pathway	Genes	q-value
Immune response (mahogany color-type mink only)	Longevity regulating pathway	*AKT3*, *ATF6B*, *CAMK4*, *CAT*, *CREB5*, *EHMT2*, *IGF1R*, *IRS1*, *PRKAA1*, *PRKAA2*, *RPTOR*, and *SOD2*	0.049
Female reproductive performance	MAPK signaling pathway	*AKT3, ATF2*, *BRAF*, *CACNB1*, *CACNB4*, *CASP3*, *DUSP3*, *DUSP6*, *EGF*, *ERBB4*, *FGF17*, *FGF18*, *FGFR2*, *FLNB*, *IKBKB*, *KIT*, *KITLG*, *MAP2K5*, *MAP3K11*, *MAP3K2*, *MYC*, *NFKB1*, *PDGFRA*, *PPP3CA*, *PPP3CC*, *RELA*, and *STK3*	0.015

## 4 Discussion

The failure to control AD by the culling strategy, immunoprophylaxis, and medical treatment resulted in the selection of AD-resilient mink based on the diagnostic tests or individual production performances (Knuuttila et al., 2009; Farid and Rupasinghe, 2016; Farid and Ferns, 2017; Farid et al., 2018). Although the phenotypic selection of AD-resilient mink is conducted in many AD-positive mink farms, the genomic architecture of AD resilience is still unclear, which might influence the effectiveness of selecting AD-resilient mink. In this study, genotypes from the Axiom Affymetrix Mink 70K panel and three methods were applied to detect the selection signatures related to immune response, general resilience, and female reproductive performance of farmed American mink under AMDV exposure. In brief, 1,611, 1,933, and 1,538 genes were annotated from the 619, 569, and 526 selection signatures detected from immune response, general resilience, and female reproductive performance traits, respectively. Although more than a thousand genes have been annotated as potential candidates for these traits, many genes, such as the identified *LOC122904335*, *LOC122905665*, and *LOC122904336* genes, were novel genes of unknown function in mink; thus, the discussions were focused on the genes with available information in the existing literature. Functional enrichment analyses revealed that some annotated genes might play an important role in the immune system process, growth, reproduction, pigmentation, sensory perception, and detection of smell.

### 4.1 Immune responses

A total of 1,611 annotated genes were related to the immune response trait by considering the whole population (Supplementary Material S2A). Some of these annotated genes were related to the immune system process (Figure 4; Tables 4 and 5). A total of 23 genes, namely, *CCL26*, *CD28*, *CGAS*, *DEF6*, *EPOR*, *FAS*, *FYB1*, *GGT1*, *HIC2*, *IL16*, *JAK3*, *MEIS1*, *MFAP3*, *PATZ1*, *RUNX2*, *SHFL*, *SIGLEC15*, *THEMIS*, *TNFRSF21*, *TOX*, *TREM2*, *YES1*, and *ZNF572*, were related to the immune system process, which might play important roles in immune-mediated responses to AMDV infection. Three genes, namely, *TNFRSF21* (chr1: 93,535,621-93,549,929 bp), *CCL26* (chr4: 10,918,021-10,922,028 bp), and *TREM2* (chr1: 87,946,316-87,950,960 bp), are related to inflammatory processes (Stubbs et al., 2010; Santer et al., 2012; Liu et al., 2020). This may be due to several inflammations, which include interstitial nephritis, myocarditis, hepatitis, splenitis, meningoencephalitis, pneumonia, glomerulonephritis, and arteritis, caused by AD infection (Jepsen et al., 2009). Four genes, *SIGLEC15* (chr3:151,209,875–151,221,497 bp), *JAK3* (chr6: 213,007,629–213,025,411 bp), *DEF6* (ch1: 117,504,375-117,525,807 bp), and *FAS* (chr2: 164,464,382-164,489,036 bp), were related to autoimmune disorders in humans (Hsu et al., 2012; García-Bermúdez et al., 2015; Serwas et al., 2019; Läubli and Varki, 2020), and AD is defined as an immune complex-mediated disorder disease in mink (Bloom et al., 1988). Three genes, *IL16* (chr13: 150,353,853-150,448,017 bp), *THEMIS* (chr1: 73,061,569-73,141,431 bp), and *CD28* (chr3: 15,437,373-15,465,940 bp), were related to T-cell proliferation (June et al., 1987; Wilson et al., 2004; Fu et al., 2009), and the *TOX* (chr4: 75,470,800-75,728,160 bp) gene was detected to be a crucial transcription factor involved in the exhaustion of CD8^+^ T cells (Seo et al., 2019). The detection of those genes might be related to the proliferation of CD8^+^ T cells after AD infection as CD8^+^ T cells were found to double in numbers during the development of AD (Aasted, 1989). The *EPOR* (chr6: 216,150,503–216,156,409 bp) gene was discovered to be associated with the production of red blood cells, and severe anemia was observed in AD-infected mink few months after infection (McGuire et al., 1979). The *FYB1* (chr1: 286,138,312–286,169,159 bp) gene was found to be related to thrombocytopenia (Levin et al., 2015), which is one of the typical symptoms of AD infection (Gordon et al., 1967). The *CGAS* (chr1: 114,736,330–114,760,223 bp) gene was related to the production of the type I interferons and activation of inflammasomes (Wang et al., 2017; Decout et al., 2021), and the increase in the number of type I interferons was observed in the host during AD infection (Jensen et al., 2003). Meanwhile, the overrepresentation tests on the annotated genes detected one significant (q < 0.05) GO:BP, adaptive immune response (GO:0002250), related to immune response, where eight genes (*IL12B*, *TNFRSF21*, *TAP1*, *JAK3*, *TAP2*, *C7*, *THEMIS*, and *C6*) were involved.

The immune-response-related genes detected in this study were different from the genes detected by a previous study (Karimi et al., 2021). Seven genes, namely, *TRAF3IP2*, *WDR7*, *SWAP70*, *TNFRSF11A*, *CBFB*, *IGF2R*, and *GPR65*, were detected and related to the immune system process, as explained by Karimi et al. (2021), and none of these genes were detected in the current study. Several potential reasons could lead to these discrepancies: 1) the use of different types of genomic data (GBS in their study vs genotypes in this study); 2) the use of different grouping methods, where kidney lesion levels and virus loads were also considered in grouping animals in their study in addition to antibody titer; 3) the different ways the animal contracted AMDV (intranasal inoculation in their study vs natural exposure in this study); and 4) the color types of studied mink (only black in their study vs multiple colors in this study).

A total of 20, 26, 19, and 9 genes were related to the immune response in black, demi, mahogany, and pastel color types, respectively (Table 3). Most of the genes detected from a single-color type were unique from the rest of the color types. For black, demi, and mahogany color types, there were few genes in common between the two color types, but no common gene was detected among all of them. For pastel, eight of nine detected genes (only *TNFRSF1B* gene was common with the black color type) were unique from the rest of the three darker color types, which might indicate that pastel color-type mink has different immune responses to AD infection compared with the other three darker color types of mink. This could be a potential reason to explain the previous finding by Ellis (1996), where the mink with lighter color types were observed to be more susceptible to the AMDV than darker mink.

The KEGG pathway analysis of annotated genes from the whole population or different color types only detected one significant KEGG pathway, the longevity regulation pathway, in mahogany color-type mink. The relationship between longevity and immune response is complex. A strong and well-functioning immune system is crucial for protecting an organism from infections and other threats, and therefore, may contribute to increased longevity (Xia et al., 2019). Furthermore, chronic inflammation and overactivation of the immune system have been linked to aging and age-related diseases, which can shorten lifespan (Rea et al., 2018). Aleutian disease is defined as an immune complex disease and can cause persistent and chronic infection in mink (Porter and Cho, 1980; Stolze and Kaaden, 1987). Thus, the detection of the longevity regulation pathway may be related to chronic infection and autoimmune disorders caused by AD.

### 4.2 General resilience

Since the general response trait used in this study was a combination of three AD resilient traits, which include growth, feeding efficiency, and pelt quality, we focused on genomic regions containing genes related to these traits. A total of 1,933 genes were related to the general resilience trait (Supplementary Material S3B). From these, several annotated genes were related to body growth. For example, *PRKAG3* (chr3: 29,115,382–29,120,999 bp), a regulatory subunit of the AMP-activated protein kinase, was detected in this study and found to be related to body growth in several livestock species including swine (Ryan et al., 2012), sheep (Ibrahim, 2015), and beef cattle (Li et al., 2012). The *PLAG1* (chr1: 58,588,578–58,696,784 bp) gene was also detected in this study. This gene is a positive regulator of insulin-like growth factor 2 (Voz et al., 2000; Van Dyck et al., 2007) that is known to affect body weight in both livestock (e.g., swine (Van Laere et al., 2003) and beef cattle (Huang et al., 2013)) and humans (Sandhu et al., 2003). The *TMEM18* (chr1: 8,940,691–9,011,524 bp) gene detected in this study has been reported to be associated with growth traits and obesity in rats (Rask-Andersen et al., 2012), cattle (Ma et al., 2012), and humans (Almén et al., 2010; Haupt et al., 2010). Meanwhile, three genes, *TPRA1* (chr6: 165,580,535-165,594,267 bp), *MCM2* (chr6: 165,600,097-165,621,492 bp), and *Tbx18* (chr1: 44,894,084-44,922,875 bp), which were all found to be related to embryo development in mice (Aki et al., 2008; Wehn and Chapman, 2010; Xu et al., 2022), were also detected in this study indicating that AD may influence the early stages of mink development, and therefore, influence growth. Meanwhile, several genes related to feed efficiency were also detected, for example, *MRAP2* (chr1: 45,464,087-45,513,205 bp) and *GLP1R* (chr1: 85,852,947-85,884,385 bp). *MRAP2* (Berruien and Smith, 2020) and *GLP1R* (Dailey and Moran, 2013) were found to play important roles in regulating appetite, and AD has been reported to cause adverse influences on the appetite of infected mink (Jensen et al., 2016). The annotated gene, *HCRTR2* (chr1: 101,039,715-101,147,858 bp), is an orexin receptor and plays an important role in feeding behavior and balance of energy metabolism (Spinazzi et al., 2006; Belkina and Denis, 2012). In addition, several annotated genes, including *ESRRG* (chr10: 14,353,012-14,953,383 bp), *LZTFL1* (chr6: 208,512,050-208,525,767 bp), and *ELOVL4* (chr1: 49,176,236-49,208,238 bp), were reported to play key roles in regulating metabolism processes (Zhang et al., 2001; Alaynick et al., 2007; Wei et al., 2018). In addition to the genes related to growth and feed efficiency, the *DCT* gene (chr5: 151,495,518-151,529,776 bp), related to pigmentation (Guyonneau et al., 2004), was also detected in this study, and this might be related to the hair depigmentation, which causes single white hairs in the fur (sprinklers) impacting the pelt quality of AD infected mink (Farid and Ferns, 2011).

### 4.3 Female reproductive performance

A total of 1,538 genes were detected from female reproductive performance traits (Figure 7; Table 6). From these, several genes, including *SLX4* (chr14: 18,318,274-18,338,014 bp), *TDRD6* (chr1: 92,984,166-92,997,290 bp), *TACR3* (chr11: 104,943,216-105,013,153 bp), *SHOC1* (chr9: 21,365,597-21,443,498 bp), *FBXW11* (chr1: 255,864,225-255,951,567 bp), *EPC2* (chr3: 82,648,215- 82,734,211 bp), GSC (chr13: 10,120,630-10,122,701 bp), and *DICER1* (chr3: 9,803,946-9,876,750 bp), were related to reproduction. *SLX4* (Hamer and de Rooij, 2018), *TDRD6* (Vasileva et al., 2009), *SHOC1* (Zhang Q. et al., 2019), and *FBXW11* (O’Doherty et al., 2018) play important roles in the development of germ cells. The gene *TACR3* plays a key role in reproductive functions, and loss-of-function mutations in this gene can lead to hypogonadotropic hypogonadism and infertility in humans (Guran et al., 2009; Topaloglu et al., 2009; Young et al., 2010). *EPC2*, *GSC*, and *DICER1* genes are all important for the development of early embryos in animals and have been related to the reproduction traits (e.g., litter size) in swine and cattle (Kaczmarek et al., 2020; Chen J. et al., 2022; Chen Z. et al., 2022; Wang et al., 2022). The reproduction-related genes detected in this study were different from the genes detected in the signature selection study for response to Aleutian disease by Karimi et al. (2021). In that study, the genes *FBXO5*, *CATSPER4*, *GOT2*, and *CatSperβ* were annotated from the candidate selection regions and related to reproductive performance, which were not detected in our study. The different genomic data, grouping methods, and population structures could be the potential reasons that lead to the differences between these studies.

The KEGG pathway analyses of annotated genes detected only one significant (q < 0.05) pathway, the MAPK signaling pathway. The MAPK signaling pathway is involved in female reproductive performance by regulating the proliferation, differentiation, and apoptosis of granulosa cells in the follicle, ultimately affecting folliculogenesis and oocyte maturation (Zhang and Liu, 2002; Sun et al., 2016; Huang et al., 2022). The MAPK pathway also plays a role in regulating luteinizing hormone secretion, which stimulates ovulation and formation of the corpus luteum (Kahnamouyi et al., 2018). Additionally, MAPK signaling has been implicated in regulating the menstrual cycle and endometrial function (Zhou et al., 2010; Makieva et al., 2018). Meanwhile, previous studies found that abnormal MAPK signaling can cause reproductive disorders (e.g., infertility and embryonic death) in swine (Prochazka et al., 2012; Prochazka and Nemcova, 2019) and cattle (Sigdel et al., 2021; Tahir et al., 2021). The detection of the MAPK signaling pathway in this study may indicate that AD infection may lead to the disorder of the MAPK signaling pathway, therefore influencing female reproductive performance.

### 4.4 Common genes and ontology terms among all three traits

A total of 16 genes, *ARHGAP19*, *COL14A1*, *DEPTOR*, *EXOSC1*, *FAM135B*, *FRAT1*, *FRAT2*, *LOC122905718*, *MMS19*, *MRPL13*, *PGAM1*, *PTCHD4*, *RRP12*, *TBX18*, *UBTD1*, and *ZDHHC16*, were common to all three studied traits. From these, five genes were related to growth in livestock species in previous studies. For example, the gene *ARHGAP19* was found to be related to body weight in yak (Jiang et al., 2022); the gene *FAM135B* was related to body weight growth in cattle (Serão et al., 2013; Seabury et al., 2017), the genes *COL14A1* (Cardoso et al., 2018) and *PTCHD4* (Doyle et al., 2020) were found to play important roles in muscle development in cattle and swine, the gene *EXOSC1* (Dall'Olio et al., 2020; Ropka-Molik et al., 2018) has been related to muscle growth, and *PGAM1* was found to relate to the development of adipose tissue (Xing et al., 2019). Meanwhile, two genes, *FAM135B* (Serão et al., 2013; Seabury et al., 2017) and *COL14A1* (de Lima et al., 2020), were also found to be related to feed efficiency in cattle. In addition, several genes were found to be related to reproduction in previous studies. *UBTD1* (Kongmanas et al., 2015), *ZDHHC16* (Uzbekova et al., 2021; Caetano et al., 2023), *RRP12* (Tiensuu et al., 2019), *MMS19* (Tsai et al., 2017), and *PGAM1* (Zhang et al., 2015) genes were found to be associated with the development of germ cells. The genes *FAM135B* and *FRAT1* were detected to be associated with the reproductive performance in swine (Zhang Z. et al., 2019) and cows (Melo et al., 2017), respectively. The Tbx18 gene was related to mouse embryonic development (Wehn and Chapman, 2010).

Two GO terms, olfactory receptor activity (GO:0004984) and detection of chemical stimulus involved in sensory perception of smell (GO:0050911), were significant (q < 0.05) among all three studied traits. This may indicate that AD may influence the sense of smell in mink, although the relationship between AD and smell has not been reported in the literature. However, reduced appetite of infected mink seems to corroborate the loss of their sense of smell because the smell is vital for mink feeding behavior (Saunders, 1988). In American mink, Adney et al. (2022) speculated that individuals experimentally infected with SARS-CoV-2 may have altered sense of smell because they observed neutrophilic infiltrate in the olfactory epithelium. Thus, future studies could assess the condition of the olfactory epithelium of AD-infected mink to determine if infection could influence their sense of smell.

## 5 Conclusion

The detection of potential signatures of selection related to the response of American mink to AD provides valuable insights into the genetic factors associated with the mink’s immune response. The genes and GO terms detected from this study would enhance the understanding of genomic architecture underlying mink’s resilience to AD and shed light on the underlying biological mechanisms involved. Meanwhile, the detection of numerous potential loci underlying the selection for responses to AD infection in this study indicated that genomic selection could be a feasible approach to reduce the adverse influence caused by AD. By incorporating the detected loci with the availability of the first Axiom Affymetrix Mink 70K panel, the mink industry could eradicate the adverse influences caused by AD by increasing the resilience of American mink to AMDV infection through genomic selection.

## Data Availability

Due to confidentiality agreements with participating farms and industries, the datasets presented in this article are not suitable for public deposition. To access the dataset used in this research, the request should be directed to the corresponding author, YM.
